# Bis(2-amino-4-methyl-1,3-thia­zole-κ*N* ^3^)dichloridocadmium(II)

**DOI:** 10.1107/S1600536808027864

**Published:** 2008-09-06

**Authors:** Lai-Jun Zhang, Xing-Can Shen, Hong Liang

**Affiliations:** a Department of Chemistry, Shangrao Normal University, Shangrao 334001, People’s Republic of China; b Key Laboratory of Medicinal Chemical Resources and Molecular Engineering, Department of Chemistry and Chemical Engineering, Guangxi Normal University, Guilin 541004, People’s Republic of China

## Abstract

In the title compound, [CdCl_2_(C_4_H_6_N_2_S)_2_], the Cd^II^ atom is coordinated by two chlorido ligands and two N atoms of the 2-amino-5-methyl-1,3-thia­zole (amtz) ligands in a slightly distorted tetra­hedral coordination geometry. Intra- and inter­molecular N—H⋯Cl hydrogen bonding stabilizes the crystal structure. A weak S⋯Cl inter­action [3.533 (2) Å] is observed between neighboring mol­ecules.

## Related literature

For general background, see: Bolos *et al.* (1999 ▶); Miodragović *et al.* (2006 ▶); Cini *et al.* (2007 ▶); Dea *et al.* (2008 ▶); Shen *et al.* (2008 ▶). For a related structure, see: Cai *et al.* (2008 ▶).
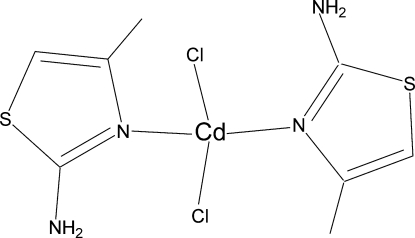


## Experimental

### 

#### Crystal data

[CdCl_2_(C_4_H_6_N_2_S)_2_]
*M* *_r_* = 411.67
Monoclinic, 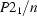

*a* = 8.7100 (17) Å
*b* = 13.190 (3) Å
*c* = 12.740 (3) Å
β = 95.19 (3)°
*V* = 1457.6 (6) Å^3^
*Z* = 4
Mo *K*α radiation
μ = 2.13 mm^−1^
*T* = 293 (2) K
0.40 × 0.25 × 0.23 mm

#### Data collection

Bruker APEXII CCD area-detector diffractometer
Absorption correction: multi-scan (*SADABS*; Bruker, 2001 ▶) *T*_min_ = 0.442, *T*_max_ = 0.612
7630 measured reflections
2595 independent reflections
2113 reflections with *I* > 2σ(*I*)
*R*_int_ = 0.027

#### Refinement

*R*[*F* ^2^ > 2σ(*F* ^2^)] = 0.027
*wR*(*F* ^2^) = 0.064
*S* = 0.98
2595 reflections
156 parameters
H-atom parameters constrained
Δρ_max_ = 0.41 e Å^−3^
Δρ_min_ = −0.39 e Å^−3^

### 

Data collection: *APEX2* (Bruker, 2004 ▶); cell refinement: *SAINT-Plus* (Bruker, 2001 ▶); data reduction: *SAINT-Plus*; program(s) used to solve structure: *SHELXTL* (Sheldrick, 2008 ▶); program(s) used to refine structure: *SHELXTL*; molecular graphics: *SHELXTL*; software used to prepare material for publication: *SHELXTL*.

## Supplementary Material

Crystal structure: contains datablocks I, global. DOI: 10.1107/S1600536808027864/xu2449sup1.cif

Structure factors: contains datablocks I. DOI: 10.1107/S1600536808027864/xu2449Isup2.hkl

Additional supplementary materials:  crystallographic information; 3D view; checkCIF report

## Figures and Tables

**Table:** 

Cd1—N2	2.246 (3)
Cd1—N1	2.248 (3)
Cd1—Cl1	2.4181 (10)
Cd1—Cl2	2.4387 (11)

**Table:** 

N2—Cd1—N1	99.70 (11)
N2—Cd1—Cl1	106.53 (8)
N1—Cd1—Cl1	116.26 (8)
N2—Cd1—Cl2	114.38 (8)
N1—Cd1—Cl2	107.19 (8)
Cl1—Cd1—Cl2	112.34 (4)

**Table 2:** Hydrogen-bond geometry (Å, °)

*D*—H⋯*A*	*D*—H	H⋯*A*	*D*⋯*A*	*D*—H⋯*A*
N3—H3*A*⋯Cl2	0.86	2.49	3.322 (4)	164
N3—H3*B*⋯Cl1^i^	0.86	2.70	3.343 (3)	133
N4—H4*A*⋯Cl1	0.86	2.44	3.276 (4)	165
N4—H4*B*⋯Cl2^ii^	0.86	2.52	3.325 (3)	157

Symmetry codes: (i) 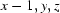; (ii) 
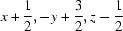
.

**Fig. 1.**
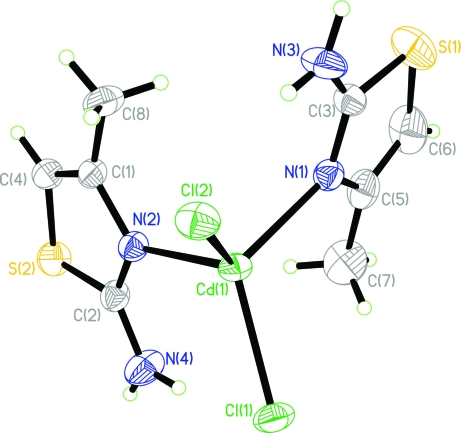
The molecular structure of the title complex. Displacement ellipsoids are drawn at the 30% probability level.

**Fig. 2.**
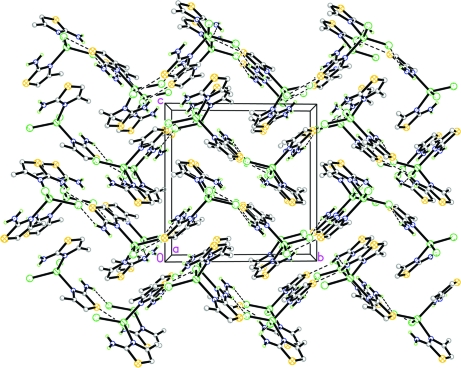
The crystal packing of the title compound viewed along the a axis in one-dimensional zigzag chain form via both the intermolecular N—H···Cl hydrogen bonds and weak S···Cl interactions which are shown by dashed lines. The intramolecular N—H···Cl hydrogen bonds and all hydrogen atoms not involved in hydrogen bonding were omitted for clarity.

**Fig. 3.**
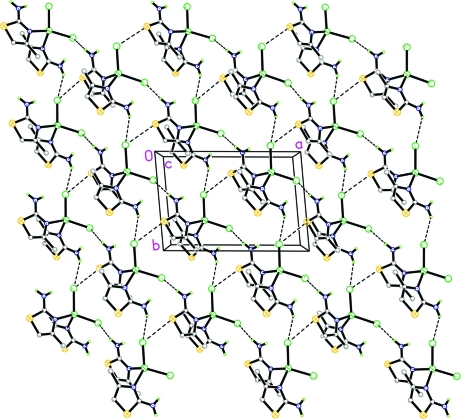
The crystal packing of the title compound viewed along the c axis, showing formation of the two-dimensional network structure via both the intermolecular N—H···Cl hydrogen bonds and weak S···Cl interactions which are denoted with dashed lines. All hydrogen atoms not involved in the intermolecular N—H···Cl hydrogen bonds were omitted for clarity.

## supplementary crystallographic information

### Comment

As one of the important S,*N*-containing-heterocycles, the 1,3-thiazole have often been regarded as a kind of pharmaceutical intermediates and constituents of many biomolecules. Higher pharmacological activities of metal-thiazole complexes than those of thiazole ligands themselves were found, which may depend on their crystal and molecular structures (Bolos *et al.* 1999; Miodragović *et al. *2006; Cini *et al.* 2007; Dea *et al.* 2008; Shen *et al.* 2008). For 2-amino-5-methyl-1,3-thiazole (amtz), however, only one Cu-containing coordination complex with definite crystal structure was reported (Bolos *et al.* 1999). Herein, our initial goal of this research is to obtain the single crystal using 2-amino-4-thiazole acetic acid (atac) as ligand. When the reaction using the raw materials such as atac and cadmium chloride hydrate [CdCl_2_.2.5(H_2_O)] in ethanol-water mixed solvents was carried out under solvothermal condition, however, atac was decarboxylized and then turn into amtz which may bind to CdCl_2_ to construct the title complex.

Fig. 1 displays the molecular structure of the title compound. The Cd^II^ atom is coordinated by two chloride anions and two N atoms of thiazole rings from two amtz ligands in a slightly distorted tetrahedral coordination geometry (Table 1) (Cai *et al.* 2008). In the crystal structure, the intramolecular N—H···Cl hydrogen bonds (Table 2) stabilize the molecular conformation, and the molecules are interconnected into a two-dimensional network structure *via* both the intermolecular N—H···Cl hydrogen bonds and weak S···Cl interactions [3.533 (2) Å]. In the crystal packing diagrams, one-dimensional zigzag chains viewed along the *a* axis and two-dimensional network structures viewed along the *c* axis can be found in Fig. 2 and in Fig. 3, respectively.

### Experimental

2-Amino-4-thiazole acetic acid (0.316 g, 2 mmol) and CdCl_2_.2.5H_2_O (0.457 g, 2 mmol) were added into 15 ml ethanol–water (1:1 volume ratio) mixed solvents and stirred for 30 min. The mixture was transferred into a Teflon-lined stainless steel vessel (25 ml). The autoclave was sealed and heated at 383 K for two days, and then autoclave was allowed to cool to room temperature in air. After isolated by filtration, the filtrate was left to stand at room temperature about one week. The brown–yellow block single crystals suitable for X-ray diffraction were obtained with the reaction yield of 30% (based on cadmium).

### Refinement

All H atoms bonded to C or N atoms were placed in geometrically calculated positions (N—H, 0.86 Å; C—H, 0.93–0.96 Å) and refined as riding with *U*_iso_(H) = 1.2*U*_eq_(C) and *U*_iso_(H) = 1.5*U*_eq_(N).

### Crystal data

**Table:**

[CdCl_2_(C_4_H_6_N_2_S)_2_]	*F*(000) = 808
*M**_r_* = 411.67	*D*_x_ = 1.885 Mg m^−^^3^
Monoclinic, *P*2_1_/*n*	Mo *K*α radiation, λ = 0.71073 Å
Hall symbol: -P 2yn	Cell parameters from 2595 reflections
*a* = 8.7100 (17) Å	θ = 2.2–25.1°
*b* = 13.190 (3) Å	µ = 2.14 mm^−^^1^
*c* = 12.740 (3) Å	*T* = 293 K
β = 95.19 (3)°	Block, brown-yellow
*V* = 1457.6 (6) Å^3^	0.40 × 0.25 × 0.23 mm
*Z* = 4	

### Data collection

**Table:**

Bruker APEXII CCD area-detector diffractometer	2595 independent reflections
Radiation source: fine-focus sealed tube	2113 reflections with *I* > 2σ(*I*)
graphite	*R*_int_ = 0.027
φ and ω scans	θ_max_ = 25.1°, θ_min_ = 2.2°
Absorption correction: multi-scan (SADABS; Bruker, 2001)	*h* = −10→10
*T*_min_ = 0.442, *T*_max_ = 0.612	*k* = −15→15
7630 measured reflections	*l* = −15→8

### Refinement

**Table:**

Refinement on *F*^2^	Primary atom site location: structure-invariant direct methods
Least-squares matrix: full	Secondary atom site location: difference Fourier map
*R*[*F*^2^ > 2σ(*F*^2^)] = 0.027	Hydrogen site location: inferred from neighbouring sites
*wR*(*F*^2^) = 0.064	H-atom parameters constrained
*S* = 0.98	*w* = 1/[σ^2^(*F*_o_^2^) + (0.0309*P*)^2^ + 0.4982*P*] where *P* = (*F*_o_^2^ + 2*F*_c_^2^)/3
2595 reflections	(Δ/σ)_max_ < 0.001
156 parameters	Δρ_max_ = 0.42 e Å^−^^3^
0 restraints	Δρ_min_ = −0.39 e Å^−^^3^

### Special details

**Table:**

Experimental. IR (KBr, cm^-1^): 3431s, 3861s, 3305s, 3205ms, 3133w, 3100w, 2978w, 2947w, 2913w, 2713w, 2346w, 1621vs, 1561*m*, 1506s, 1438ms, 1380ms, 1357s, 1147*m*, 1112s, 1033*m*, 843w, 738*m*, 703*m*, 637*m*, 606*m*, 478*m*.
Geometry. All e.s.d.'s (except the e.s.d. in the dihedral angle between two l.s. planes) are estimated using the full covariance matrix. The cell e.s.d.'s are taken into account individually in the estimation of e.s.d.'s in distances, angles and torsion angles; correlations between e.s.d.'s in cell parameters are only used when they are defined by crystal symmetry. An approximate (isotropic) treatment of cell e.s.d.'s is used for estimating e.s.d.'s involving l.s. planes.
Refinement. Refinement of *F*^2^ against ALL reflections. The weighted *R*-factor *wR* and goodness of fit *S* are based on *F*^2^, conventional *R*-factors *R* are based on *F*, with *F* set to zero for negative *F*^2^. The threshold expression of *F*^2^ > σ(*F*^2^) is used only for calculating *R*-factors(gt) *etc*. and is not relevant to the choice of reflections for refinement. *R*-factors based on *F*^2^ are statistically about twice as large as those based on *F*, and *R*- factors based on ALL data will be even larger.

### Fractional atomic coordinates and isotropic or equivalent isotropic displacement parameters (Å^2^)

**Table:**

	*x*	*y*	*z*	*U*_iso_*/*U*_eq_	
Cd1	0.79522 (3)	0.902858 (19)	0.78981 (2)	0.04161 (10)	
C1	0.6219 (4)	0.7247 (3)	0.6506 (3)	0.0454 (9)	
C2	0.8598 (4)	0.7492 (3)	0.6053 (3)	0.0468 (9)	
C3	0.4937 (4)	1.0397 (3)	0.7472 (3)	0.0510 (9)	
C4	0.6202 (4)	0.6597 (3)	0.5703 (3)	0.0535 (10)	
H4	0.5368	0.6187	0.5480	0.064*	
C5	0.6514 (5)	1.0670 (3)	0.6215 (3)	0.0621 (11)	
C6	0.5336 (7)	1.1274 (4)	0.5873 (4)	0.0879 (16)	
H6	0.5319	1.1667	0.5267	0.105*	
C7	0.7954 (6)	1.0484 (4)	0.5704 (4)	0.0891 (16)	
H7A	0.7925	1.0860	0.5057	0.134*	
H7B	0.8043	0.9774	0.5557	0.134*	
H7C	0.8824	1.0698	0.6167	0.134*	
C8	0.4931 (4)	0.7448 (3)	0.7171 (3)	0.0659 (12)	
H8A	0.4101	0.6985	0.6983	0.099*	
H8B	0.4573	0.8132	0.7058	0.099*	
H8C	0.5288	0.7359	0.7900	0.099*	
Cl1	1.06487 (10)	0.94872 (8)	0.79854 (9)	0.0612 (3)	
Cl2	0.71199 (11)	0.86235 (8)	0.96262 (7)	0.0551 (3)	
N1	0.6277 (3)	1.0170 (2)	0.7149 (2)	0.0443 (7)	
N2	0.7612 (3)	0.7769 (2)	0.6713 (2)	0.0427 (7)	
N3	0.4407 (3)	1.0036 (3)	0.8341 (3)	0.0649 (9)	
H3A	0.4957	0.9620	0.8735	0.078*	
H3B	0.3512	1.0216	0.8509	0.078*	
N4	1.0026 (4)	0.7866 (3)	0.6046 (3)	0.0710 (11)	
H4A	1.0348	0.8321	0.6497	0.085*	
H4B	1.0621	0.7651	0.5591	0.085*	
S1	0.38830 (17)	1.12403 (11)	0.66679 (12)	0.0917 (4)	
S2	0.79042 (12)	0.66122 (8)	0.51342 (8)	0.0607 (3)	

### Atomic displacement parameters (Å^2^)

**Table:**

	*U*^11^	*U*^22^	*U*^33^	*U*^12^	*U*^13^	*U*^23^
Cd1	0.03346 (15)	0.04928 (17)	0.04276 (17)	−0.00234 (11)	0.00714 (11)	−0.00173 (12)
C1	0.041 (2)	0.041 (2)	0.052 (2)	−0.0037 (16)	−0.0011 (17)	0.0042 (18)
C2	0.050 (2)	0.043 (2)	0.048 (2)	−0.0025 (17)	0.0102 (17)	−0.0039 (17)
C3	0.043 (2)	0.047 (2)	0.062 (3)	0.0040 (17)	−0.0009 (19)	−0.0053 (19)
C4	0.048 (2)	0.048 (2)	0.062 (3)	−0.0031 (18)	−0.0063 (19)	−0.007 (2)
C5	0.080 (3)	0.049 (2)	0.056 (3)	−0.008 (2)	0.002 (2)	0.012 (2)
C6	0.118 (4)	0.069 (3)	0.072 (3)	0.009 (3)	−0.013 (3)	0.030 (3)
C7	0.104 (4)	0.099 (4)	0.070 (3)	−0.008 (3)	0.034 (3)	0.026 (3)
C8	0.045 (2)	0.075 (3)	0.079 (3)	−0.014 (2)	0.015 (2)	−0.011 (2)
Cl1	0.0355 (5)	0.0727 (7)	0.0757 (7)	−0.0109 (5)	0.0062 (5)	−0.0115 (6)
Cl2	0.0497 (5)	0.0708 (6)	0.0466 (5)	0.0022 (5)	0.0140 (4)	0.0077 (5)
N1	0.0445 (17)	0.0412 (17)	0.0470 (18)	−0.0030 (14)	0.0030 (14)	0.0039 (14)
N2	0.0429 (16)	0.0425 (16)	0.0436 (16)	−0.0034 (13)	0.0084 (13)	−0.0036 (14)
N3	0.0409 (18)	0.083 (2)	0.073 (2)	0.0146 (17)	0.0172 (17)	0.008 (2)
N4	0.059 (2)	0.078 (2)	0.081 (3)	−0.0172 (19)	0.0366 (19)	−0.028 (2)
S1	0.0823 (9)	0.0895 (9)	0.1004 (11)	0.0353 (7)	−0.0071 (8)	0.0189 (8)
S2	0.0642 (7)	0.0591 (6)	0.0589 (7)	0.0000 (5)	0.0061 (5)	−0.0178 (5)

### Geometric parameters (Å, °)

**Table:**

Cd1—N2	2.246 (3)	C5—C6	1.340 (6)
Cd1—N1	2.248 (3)	C5—N1	1.392 (5)
Cd1—Cl1	2.4181 (10)	C5—C7	1.485 (6)
Cd1—Cl2	2.4387 (11)	C6—S1	1.691 (6)
C1—C4	1.333 (5)	C6—H6	0.9300
C1—N2	1.399 (4)	C7—H7A	0.9600
C1—C8	1.490 (5)	C7—H7B	0.9600
C2—N2	1.308 (4)	C7—H7C	0.9600
C2—N4	1.338 (4)	C8—H8A	0.9600
C2—S2	1.718 (4)	C8—H8B	0.9600
C3—N1	1.308 (4)	C8—H8C	0.9600
C3—N3	1.326 (5)	N3—H3A	0.8600
C3—S1	1.720 (4)	N3—H3B	0.8600
C4—S2	1.708 (4)	N4—H4A	0.8600
C4—H4	0.9300	N4—H4B	0.8600
			
N2—Cd1—N1	99.70 (11)	C5—C7—H7B	109.5
N2—Cd1—Cl1	106.53 (8)	H7A—C7—H7B	109.5
N1—Cd1—Cl1	116.26 (8)	C5—C7—H7C	109.5
N2—Cd1—Cl2	114.38 (8)	H7A—C7—H7C	109.5
N1—Cd1—Cl2	107.19 (8)	H7B—C7—H7C	109.5
Cl1—Cd1—Cl2	112.34 (4)	C1—C8—H8A	109.5
C4—C1—N2	114.2 (3)	C1—C8—H8B	109.5
C4—C1—C8	126.5 (3)	H8A—C8—H8B	109.5
N2—C1—C8	119.3 (3)	C1—C8—H8C	109.5
N2—C2—N4	124.4 (3)	H8A—C8—H8C	109.5
N2—C2—S2	114.6 (3)	H8B—C8—H8C	109.5
N4—C2—S2	121.0 (3)	C3—N1—C5	111.5 (3)
N1—C3—N3	124.6 (3)	C3—N1—Cd1	125.7 (3)
N1—C3—S1	113.8 (3)	C5—N1—Cd1	122.7 (3)
N3—C3—S1	121.5 (3)	C2—N2—C1	110.4 (3)
C1—C4—S2	111.6 (3)	C2—N2—Cd1	125.8 (2)
C1—C4—H4	124.2	C1—N2—Cd1	123.4 (2)
S2—C4—H4	124.2	C3—N3—H3A	120.0
C6—C5—N1	113.0 (4)	C3—N3—H3B	120.0
C6—C5—C7	127.4 (4)	H3A—N3—H3B	120.0
N1—C5—C7	119.5 (4)	C2—N4—H4A	120.0
C5—C6—S1	112.5 (4)	C2—N4—H4B	120.0
C5—C6—H6	123.8	H4A—N4—H4B	120.0
S1—C6—H6	123.8	C6—S1—C3	89.2 (2)
C5—C7—H7A	109.5	C4—S2—C2	89.09 (18)

### Hydrogen-bond geometry (Å, °)

**Table:**

*D*—H···*A*	*D*—H	H···*A*	*D*···*A*	*D*—H···*A*
N3—H3A···Cl2	0.86	2.49	3.322 (4)	164.
N3—H3B···Cl1^i^	0.86	2.70	3.343 (3)	133.
N4—H4A···Cl1	0.86	2.44	3.276 (4)	165.
N4—H4B···Cl2^ii^	0.86	2.52	3.325 (3)	157.

Symmetry codes: (i) *x*−1, *y*, *z*; (ii) *x*+1/2, −*y*+3/2, *z*−1/2.
